# The stability of colour-emotion associations across colour presentation modes and experimental settings

**DOI:** 10.3758/s13423-026-02881-z

**Published:** 2026-04-20

**Authors:** Abdulrahman S. Al-rasheed, Christine Mohr, Déborah Epicoco, Domicele Jonauskaite

**Affiliations:** 1 Department of Psychology, King Saud University, Riyadh, Kingdom of Saudi Arabia; 2 Institute of Psychology, University of Lausanne, CH-1015 Lausanne, Switzerland; 3 School of Social Work, University of Applied Sciences and Arts of Western Switzerland (HETSL | HES-SO), Lausanne, Switzerland

**Keywords:** Colour, Affect, Cross-modal correspondences, Online experiments

## Abstract

Colour-emotion associations seem consistent across cultures, but the question remains whether these associations depend on actual colour perception. We examined whether colour-emotion associations are invariant to colour presentation mode (colour terms vs. colour patches) and experimental setup (laboratory vs. online). In a 2 × 2 between-subjects design, 873 Arabic speakers in Saudi Arabia associated 20 emotion concepts with 11 basic colour categories presented as terms or patches, either in controlled laboratory conditions or online. Participants self-selected focal colours online, while we used pre-selected colours in the laboratory. The most frequent association was red-love (61.5%), with distinct patterns for warm colours (associated with high arousal) and cool colours (associated with low arousal). The correlation analyses revealed remarkably similar patterns of colour-emotion associations across all four conditions (all *r* > .90, *p* < .001). While participants associated more positive emotions in the laboratory than online, no other significant differences were found for valence, arousal, or power biases between the experimental conditions. There were, however, differences for the number of associated emotions (higher online and for colour patches) and for emotion intensity (higher for colour terms), suggesting that participants exhibited somewhat different behaviours depending on the testing location and colour presentation mode. Overall, the patterns of colour-emotion associations seem largely independent of colour presentation mode and experimental setup, highlighting that these associations likely operate on an abstract representational level. Our results validate the use of both laboratory and online methodologies for studying colour-emotion associations, enabling more diverse and representative cross-cultural research.

**Supplementary Information:**

The online version contains supplementary material available at https://doi.org/10.3758/s13423-026-02881-z.

## Introduction

Throughout history, colour experiences have been thought to be a source of profound emotions (see a historical review in Elliot, 2019). Accordingly, red should evoke arousing emotions, blue should be calming, and yellow positively exciting. While modern studies showed limited evidence on colours influencing felt emotions (e.g., Al-Ayash et al., 2016; Weijs et al., 2023; Wilms & Oberfeld, 2018; see Jonauskaite et al., 2026, for an overview), this does not mean that colours and emotions are unrelated. Empirical research has shown systematic and consistent colour-emotion correspondences (Jonauskaite & Mohr, 2025), many of which hold across cultures and individuals (Adams & Osgood, 1973; Hupka et al., 1997; Jonauskaite, Abu-Akel et al., 2020a, Jonauskaite et al., 2024; Ou et al., 2018).

People can decide on such associations without seeing the colour itself, because systematic colour-emotion correspondences occur with colour terms (e.g., Hupka et al., 1997; Sutton & Altarriba, 2016; Wexner, 1954) and colour patches (e.g., Fugate & Franco, 2019; Kaya & Epps, 2004; Valdez & Mehrabian, 1994). While only a few studies directly compared colour-emotion correspondences for colour terms and patches using comparable methodologies (Jonauskaite et al., 2021, Jonauskaite, Parraga et al., 2020b; Wang et al., 2014; Xu et al., 2024), their results showed high similarities between the colour presentation modes for most colour categories in French speakers (Jonauskaite, Parraga et al., 2020b), Spanish and Mandarin Chinese speakers (Xu et al., 2024), and non-colour-blind and colour-blind French speakers (Jonauskaite et al., 2021).

Despite the overall similarities, these studies also found some exceptions. That is, term-patch correlations were the lowest for (i) purple for French speakers (Jonauskaite, Parraga et al., 2020b), (ii) green and purple for colour-blind French speakers (Jonauskaite et al., 2021), (iii) white for non-colour-blind French speakers (Jonauskaite et al., 2021), (iv) blue, purple, and light purple for Spanish speakers (Xu et al., 2024), and (v) purple and light purple for Mandarin Chinese speakers (Xu et al., 2024). Black also stood out, as it was associated with more negative emotions when presented as a term than a patch in French speakers (Jonauskaite, Parraga et al., 2020b). Finally, Wang and colleagues (2014) used red and blue as priming stimuli once as patches and once as terms while participants had to make decisions about affective words (word vs. pseudoword). Their Chinese participants showed dampened affective responses when both colours were presented as terms rather than patches, with red yielding both positive and negative connotations as a patch but only positive connotations as a term, and blue yielding positive connotations as a patch and no affective connotations as a term.

To assess whether colour-emotion correspondences are, indeed, invariant across different presentation modes, we considered studies using either colour terms or colour patches. We also took into account whether the results were derived from either laboratory or online settings. Online testing has become increasingly popular because it allows researchers to reach larger and more diverse populations (Gvirtz & Sabherwal, 2024). However, conducting colour research online presents challenges, as researchers have limited control over stimulus presentation and the testing environment (Epicoco et al., 2024; Woods et al., 2015). To address these challenges, we worked with a new two-step approach for online colour presentation. In step one, participants self-selected representative examples for each colour category. In step two, they made emotion associations with their self-selected colours. Additionally, participants self-calibrated their screens using a perception-based task (see Epicoco et al., 2024). This approach ensures that each colour category is represented by appropriate physical colours.

We collected data from four independent groups of Arabic speakers in Saudi Arabia. We always worked with the 11 basic colour categories RED, ORANGE, YELLOW, GREEN, BLUE, PURPLE, PINK, BROWN, GREY, WHITE, and BLACK. For the 11 colour terms and 20 emotion terms, we used the same Arabic terms as used before (Al-rasheed et al., 2011; Jonauskaite, Abu-Akel et al., 2020a). For colour patches, we used pre-selected focal colour patches under controlled conditions in the laboratory (Al-rasheed, 2014). For the online testing, we used participant self-selected colours, obtained in the two-step approach.

If indeed colour-emotion correspondences are abstract and depend little on the colour presentation mode, we would expect colour-emotion correspondences to be very similar, whether shown as patches or terms, in the laboratory or online, as pre-selected or self-selected colours. Based on previous studies using this methodology (Jonauskaite, Parraga et al., 2020b, Jonauskaite et al., 2021; Xu et al., 2024), we could also expect colour terms to yield stronger emotion correspondences than colour patches. To this end, we compared the patterns of discrete colour-emotion correspondences, affective biases (i.e., valence, arousal, and power), broadness (i.e., the number of emotions associated with colours), and emotion intensity. We expected the patterns of colour-emotion associations to have high correlations across the four conditions. We also expected affective biases, broadness, and intensity to be invariant of condition.

## Material and method

### Participants

We recruited 873 participants (405 men, one other gender, one did not wish to respond). Their mean age was 23.9 years (*SD* = 9.36 years). Younger participants were recruited from the student population of King Saud and Prince Norah Universities, Riyadh, Saudi Arabia. Older participants were recruited through personal contact likely including individuals outside of the university setting. None of the participants was colour-blind by self-report when tested online or according to the City University Test (Fletcher, 1980) when tested in the laboratory. Participants were native Arabic speakers and completed the study in Arabic. Participation was voluntary; some participants received monetary compensation (20 SAR). The study was conducted in accordance with the principles of the Declaration of Helsinki (World Medical Association, 2025) that are relevant to behavioural experiments. It was approved by the ethics committee (C_SSP_032020_00003). Participants took part in one of the four between-subjects conditions (see relevant demographic information in Table 1).

**Table 1:** Demographic information for our participants

				Number of participants				Age (in years)		
Experiment	Set-up condition	Colour presentation mode	Stimulus type	Total	Women	Men	Other	Mean	*SD*	Range
Experiment 1	Laboratory	Colour terms	Pre-selected	210	97	113	0	20.9	4.0	18–45
Experiment 2	Laboratory	Colour patches	Pre-selected	224	105	119	0	21.0	2.6	18–39
Experiment 3	Online	Colour terms	Pre-selected	200	132	67	1	32.1	15.1	16–85
Experiment 4	Online	Colour patches	Self-selected	239	132	106	1	22.5	5.9	18–54

*Note*. Age variables are presented in years. In Experiment 4, we retained 217 (92 men, one other) participants, following the exclusion process described in the *Data selection and cleaning* section

### Emotion stimuli

**Fig. 1:**
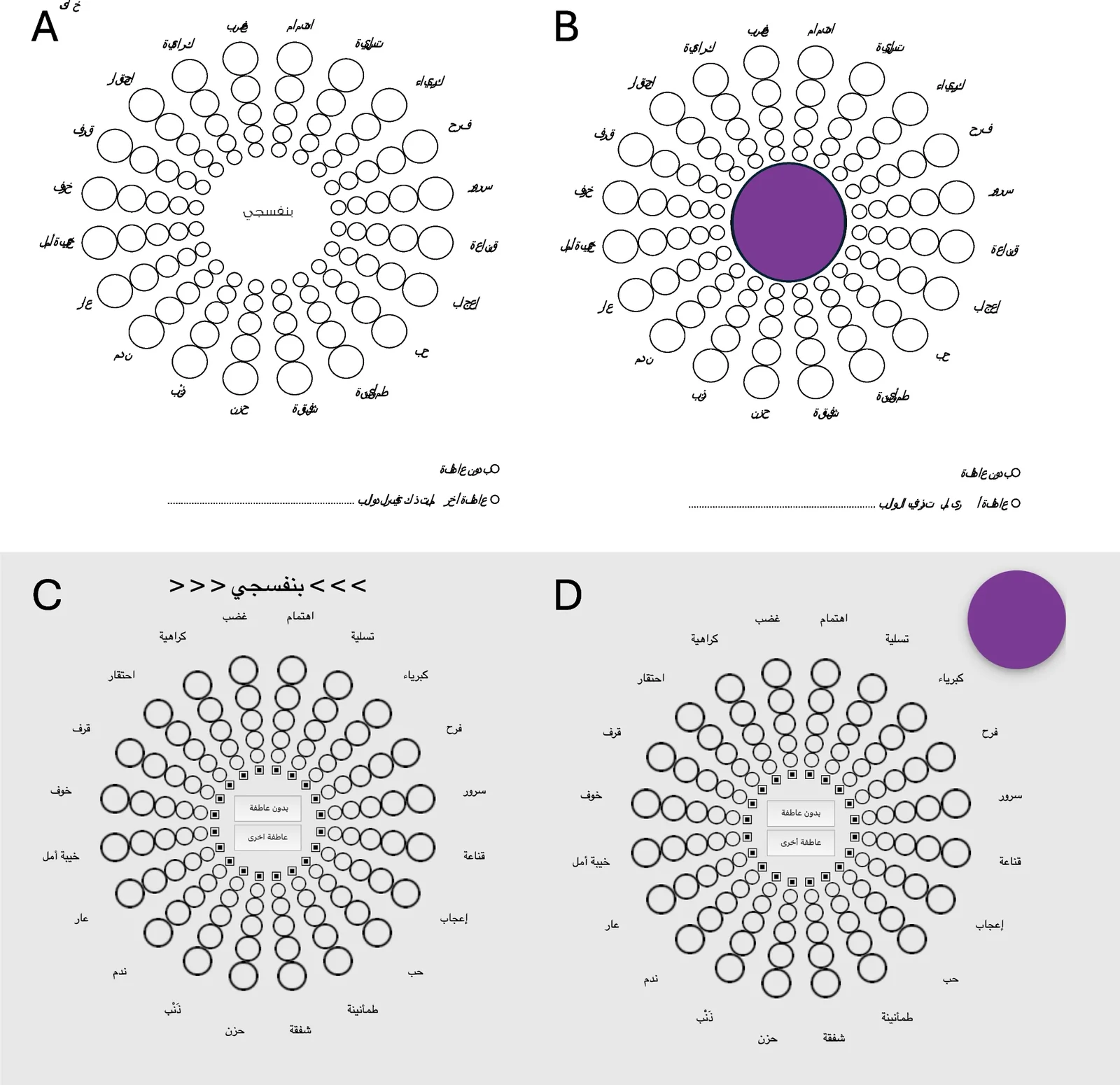
The Geneva Emotion Wheel (GEW) used to assess colour-emotion associations. (**A**) In Experiment 1, participants saw a colour term (e.g., *purple*) written in the centre of the wheel. (**B**) In Experiment 2, participants saw a pre-selected colour patch glued in the centre of the wheel. (**C**) In Experiment 3, participants saw a colour term (e.g., *purple*) written above the wheel. (**D**) In Experiment 4, participants saw their self-selected colour patch on the right side of the wheel. In Experiments 1 and 2 (**A** and **B**), at the bottom of the page, participants could choose “No emotion” or “Different emotion”, in which case they would name a different emotion. In Experiments 3 and 4 (**C** and **D**), those options appeared in the centre of the wheel, and a different emotion could be written in a pop-up window. The GEW appeared on the white sheet of paper in Experiments 1 and 2, and on a grey background in online Experiments 3 and 4. See Table 2 for the emotion terms in English and in Arabic; see Table 3 for the colour terms in English and in Arabic

We used a slightly modified version of the Geneva Emotion Wheel (GEW 3.0; Scherer, 2005; Scherer et al., 2013), which is a user-friendly self-report measurement tool of emotion. The GEW consists in 20 discrete emotion concepts (e.g., *joy*, *sadness*, etc., see Fig. 1 and Table 2) arranged in a circle along the valence and power axes. Emotion concepts that are similar in valence (left: positive, right: negative) and power (top: high power, bottom: low power) are placed next to each other. Each emotion has five circles extending from the centre to the periphery of the wheel, representing the increase of emotion intensity, with larger circles representing higher emotion intensities.

**Table 2:** Emotion concepts in English, and their Arabic translations used in the study

English	Arabic	Valence	Arousal	Power
Interest	اهتمام	Positive	Low	High
Amusement	تسلية	Positive	High	High
Pride	كبرياء	Positive	Low	High
Joy	فرح	Positive	High	High
Pleasure	سرور	Positive	High	High
Contentment	قناعة	Positive	Low	Low
Admiration	إعجاب	Positive	High	Low
Love	حب	Positive	High	Low
Relief	طمأنينة	Positive	Low	Low
Compassion	شفقة	Positive	Low	Low
Sadness	حزن	Negative	Low	Low
Guilt	ذَنْب	Negative	High	Low
Regret	ندم	Negative	Low	Low
Shame	عار	Negative	High	Low
Disappointment	خيبة أمل	Negative	Low	Low
Fear	خوف	Negative	High	High
Disgust	قرف	Negative	Low	High
Contempt	احتقار	Negative	Low	High
Hate	كراهية	Negative	High	High
Anger	غضب	Negative	High	High

These 20 emotion concepts can be grouped in terms of the underlying affective dimensions of valence, arousal, and power (see Table 2)*.* The affective loadings have been derived in a previous study, conducted in 34 populations coming from 27 countries, speaking 28 languages (Fontaine et al., 2007, 2013; Soriano et al., 2013). The same loadings have been used previously in colour-emotion research (Jonauskaite et al., 2021, Jonauskaite, Parraga et al., 2020b; Uusküla et al., 2023).

### Colour stimuli

We used 11 Arabic basic colour categories as stimuli: RED, ORANGE, YELLOW, GREEN, BLUE, PURPLE, PINK, BROWN, GREY, WHITE, and BLACK (see Table 3). These basic colour categories were presented either as terms or as patches depending on the experiment (see Table 1).

**Table 3:** Colour stimuli used in the study

			CIE coordinates								Colour term (Arabic)
Colour category	Color-Aid code		*Y* (cd/m^2^)	*x*	*y*	L*	a*	b*	C*	h*	
Red	ROR	HUE	77.92	0.5483	0.3232	44.76	55.67	30	63.25	28.31	أحمر
Orange	OYO	HUE	137.00	0.5520	0.3776	57.72	48.95	56.65	74.93	49.12	برتقالي
Yellow	Y	HUE	302.58	0.4571	0.4801	79.53	0.12	83.51	83.52	89.93	أصفر
Green	GYG	HUE	98.06	0.2477	0.4571	49.69	−48.48	18.24	51.84	159.30	أخضر
Blue	B	T1	99.49	0.1974	0.2184	49.80	−4.81	−39.48	39.77	263.04	أزرق
Purple	RV	HUE	59.59	0.4083	0.2427	39.91	50.45	−9.21	51.36	349.54	بنفسجي
Pink	R	T4	226.50	0.4049	0.3262	70.69	34.60	13.40	37.10	21.16	وردي
Brown	RO	S3	39.92	0.3945	0.3526	32.20	11.52	11.08	15.99	43.54	بوني
White	WHITE	-	418.48	0.3205	0.3395	90.00	−1.14	5.16	5.29	103.19	أبيض
Grey	GRAY	8	26.49	0.3122	0.3271	26.56	0.19	−0.22	0.63	354.79	رصاصي
Black	BLACK	-	13.00	0.3198	0.3340	17.82	0.31	1.01	1.06	73.07	أسود

*Note*. Colour terms, written in Arabic script, were used in Experiments 1 and 3. Standard Color Aid Corporation (CAC-220) colour samples were used in Experiment 2 in the laboratory, while participants selected colours themselves in Experiment 4. The colorimetric values of the Color-Aid colour samples were determined in a different study (Jonauskaite, et al., 2026). We present the colorimetric values in Commission internationale de l'éclairage (CIE) CIE *xyY*, CIE *Lab*, and CIE *LCh* colour systems, where the hue angle (h*) is specified in degrees (see the colours visually displayed in Fig. A1)

#### Experiment 1: Colour terms in the laboratory

The colour stimuli were presented as colour terms written in black ink in Arabic (see Fig. 1A and Table 3). They have been determined in previous studies (Al-rasheed, 2010, 2014; Al-rasheed et al., 2011) and appeared in the centre of the GEW.

#### Experiment 2: Colour patches in the laboratory

The colour stimuli were shown as physical colour patches, representing the best examples (i.e., focal colours) of each Arabic basic colour category, derived in previous colour-naming experiments (see Al-rasheed, 2010; Al-rasheed et al., 2011). Colour patches had been selected from the Standard set of the Color-Aid Corporation (i.e., CAC-220; https://coloraid.com/standardset220colors.aspx). The patches were placed in the middle of the GEW (see Fig. 1B). See Table 3 and Fig. A1 for the corresponding Color-Aid codes. Their colorimetric values were determined in a different study (Jonauskaite, et al., 2026) measuring the colour samples in two independent laboratories under the D-65 illumination, with 45/0 degree geometry, 2° observer angle, using PR-650 spectroradiometer and Konica Minolta CS-150 devices (averages of four measurements per colour sample).

#### Experiment 3: Colour terms online

We used the same colour terms, written in black ink in Arabic, as in the laboratory, but placed them above the GEW (see Fig. 1C and Table 3).

#### Experiment 4: Colour patches online

Participants selected their own best examples (i.e., their focal colours) for each Arabic colour category by using a colour picker, designed to facilitate the selection of target colours (see Epicoco et al., 2024; Jonauskaite et al., 2016). The colour picker starts with nine different colour patches, from which participants choose a colour which is the closest to their target colour. After the first selection, their chosen colour appears in the centre and is surrounded by eight other colour patches, slightly varying in hue, chroma, and lightness. Participants can then tweak their colour choice by clicking on the surrounding colour patches and changing the hue, lightness, or chroma of their chosen colour. Alternatively they can stay with their chosen colour by again clicking on the central patch. With each iteration, the difference between the central patch and the surrounding colour patches is reduced. Once participants are satisfied with their colour choice, they single out the central patch by clicking once or several times on it. At that point, they can continue tweaking their colour choice by clicking again on the central patch or restart the colour selection completely by clicking on the button “Choose colour again”. Alternatively, if they are satisfied with their colour choice, they can move onto the next colour selection. Their final colour choice is recorded in the RGB colour system and then converted to the *CIE* *LCh* colour system (see *Data preparation, *Experiment 4*: Colour patches online*). We also recorded the number of clicks and time taken to reach the final colour.

During the actual experiment, these personal colour patches were shown on the right-hand side, next to the GEW (see Fig. 1D). The natural reading direction in Arabic is right-to-left. Thus, our participants were likely to first look at the colour patch and then move to the GEW to make emotion associations. In other versions of this survey, for left-to-right languages (e.g., English), these colour patches are placed on the left-hand side.

### Procedure

#### Experiments 1 and 2: In the laboratory

Both experiments were conducted in Arabic and participants were tested individually by four trained experimenters. Male experimenters were assigned to male participants, and female experimenters were assigned to female participants, in compliance with the local regulations. Both experiments were conducted in two laboratories at the Department of Psychology, equipped for experimental studies on vision and colour perception. Both laboratories were lit by a ceiling daylight fixed luminaire lamp (OPPLE: MX586-83W, RV 127V~60Hz, Lamp: YH83-T9/2C). In both experiments, participants gave their informed consent, and the experimenters tested their colour vision. Afterwards, they received task instructions in a written format and completed the demographic information, all with paper and pencil. Then, participants performed the emotion-association task with either colour terms or colour patches, using paper and pencil, after which they were thanked, remunerated, and fully debriefed. The entire experiment took about 15 min to complete.

#### Colour terms condition (Experiment 1)

The task was printed on 11 sheets of A4 paper size (portrait: 297 mm height x 210 mm width), placed on a table covered with a grey cloth. On each sheet, there was the GEW (Fig. 1A), with one of the 11 colour terms written in the centre of it (see Table 3). Participants were asked to associate the GEW emotion concepts with the given colour term by shading one of the five circles of increasing size on the GEW. They could choose one, several, or none of the GEW emotion concepts. By choosing the size of the circles, participants also evaluated emotion intensities. If they had no associated emotion with the colour term, they left the circles unshaded. They could also explicitly indicate that they did have any associated emotion by ticking a relevant option at the bottom of the page. There, they could also name a different emotion they associated with the given colour term.

#### Colour patches condition (Experiment 2)

The experimental task was identical to the colour terms condition, with the exception that colour patches were used as stimuli (see the stimuli in Table 3 and Fig. A1 in the Online Supplementary Material (OSM)). These colour patches were also placed in the centre of the GEW and had a diameter of 46 mm (Fig. 1B).

#### Experiments 3 and 4: Online

Both experiments were part of the International Colour-Emotion Association Survey. The latter was originally designed with colour terms and samples of data were published previously (Griber et al., 2019; Jonauskaite, 2024; Jonauskaite, Abdel-Khalek et al., 2019a, Jonauskaite, Dael et al., 2019b, Jonauskaite, Wicker et al., 2019c, Jonauskaite, Abu-Akel et al., 2020a, Jonauskaite, Parraga et al., 2020b, Jonauskaite et al., 2024; Ram et al., 2020; Uusküla et al., 2023). Version 1 (v.1) of this survey was also used in Experiment 3. More recently, the survey had been redesigned to accommodate research with colour patches (v.2). This is the first publication of the v.2 of the International Colour-Emotion Association Survey (Experiment 4).

#### Colour terms condition (Experiment 3)

The v.1 of the survey started by stating its main goal and ethical information (e.g., confidential responses, informed consent). Participants consented by clicking on the “Let’s go” button. The following pages instructed participants on the task and the use of the GEW. To ensure comprehension, participants performed a practice trial, for which we used the colour term beige (بيج), which does not appear later in the task. Importantly, they had to correct the choice of Peter, a fictional character. Peter had made a mistake when choosing emotion concepts he thought were associated with beige. After participants successfully corrected Peter’s example, they proceeded to the main part of the survey.

In the main part of the survey, participants saw 11 basic colour terms^1^ on top of the GEW, in a randomised order (Fig. 1C). They were asked to associate one, several, or none of the GEW emotions with each colour term. They could also indicate a different emotion in a pop-up window. When making associations, participants also evaluated emotion intensities, by choosing one of the five circles of increasing size, linked with the chosen emotion (see Fig. 1). At the end of the survey, participants reported their demographic information, i.e., (i) year of birth, (ii) gender, (iii) if they were colour blind, (iv) whether colour was important in their lives, (v) native language, and (vi) the fluency of the survey language (here, Arabic). For all the demographic questions, a “Do not want to answer” option was available. On the last page, participants were thanked and presented with a graphical result of colour-emotion associations from a previous related study. On average, participants took 12.8 min to complete the survey.

#### Colour patches condition (Experiment 4)

The v.2 of the survey also started with an informed consent page and information about the goals of the survey. Participants who consented to the study clicked on the “Let’s go button”. Participants were then asked to adjust their screens to make sure they (i) have comfortable light conditions; (ii) view their screen straight ahead so that they have a full view of the screen and can see colours well; (iii) disable any blue light filters they may use (e.g., Night Shift, flux, similar); and (iv) have their webpage content set to 100% (i.e., they have not zoomed in or zoomed out of the webpage). Participants reported whether they could follow the screen calibration by selecting one of the three options: ‘Yes’, ‘No’, or ‘I don’t know how to do it’. If they did not select the option ‘Yes’, they were asked to explain why they had not been able to follow the instructions (we only analysed data from participants who said ‘Yes’).

The following page explained the structure of the survey, which consisted of three parts. In Part 1, participants were asked to perform the screen calibration routine, which was recommended but optional, lasting approximately 5 min (see a detailed description in Epicoco et al., 2024). Participants completed the screen calibration task four times for each of the three colour channels (Red, Green, and Blue). Trials were presented in a randomised order. Once finished, they proceeded to Part 2 of the experiment.

In Part 2, participants chose colours that best represented given colour terms, using the colour picker (see Experiment 4: Colour patches online). To make sure that participants understood the functioning of the colour picker, they first selected the best example for the colour *beige* (not recorded). Then, they selected their favourite and least favourite colours (not analysed here) and finally proceeded to the selection of the best examples of the 11 basic colour terms.^2^ The basic colour terms appeared one at a time in a randomised order above the colour picker and participants used the latter to select what they thought was the best example of each colour term. After having made their choices for all 11 colours, they saw all of their 11 colour selections, vertically presented on the same page, and could change any of them individually or re-start Part 2 entirely. They were able to change as many colour selections as they wished by using the same colour picker and proceeded to Part 3 once they were fully satisfied with their colour choices.

In Part 3, they associated emotion concepts with their chosen colours. Similar to v.1, after correcting the comprehension task (Peter’s choices), participants were randomly presented with the 11 colour patches they had chosen in Part 2 (Fig. 1D). They were asked to associate one, several, or none of the GEW emotion concepts and rate their intensities (see Fig. 1). The order in which the colour patches were presented was different from the order in which participants had selected the best examples for the corresponding colour terms.

At the end of the experiment, participants reported demographic information analogous to that in Experiment 3, with the following additional information: All the languages they speak and their fluency on a scale from 1 to 8; County, region, or state of residence; Nearest town; and the kind of environment they live in (urban or rural).

On the last page of the survey, participants were thanked and presented with a graphical result of colour-emotion associations from a previous related study (Jonauskaite, Abu-Akel et al., 2020a, which can also be seen in Mohr & Jonauskaite, 2022, Fig. 1). On average, participants took 31.4 min to complete the survey.

### Data selection and cleaning

#### Experiment 3: Colour terms online

As data came from a larger ongoing international online survey, we had pre-selected participants (i) who completed the survey in Arabic; (ii) whose native language was Arabic; and (iii) who originally came from Saudi Arabia. We further had filtered out participants who were colour-blind by self-report as well as those who finished the survey very quickly (under 3 min) or took very long (over 90 min) to complete the survey. The latter steps ensured participant engagement. Data from most of the Experiment 3 participants have been published in previous cross-cultural investigations (Jonauskaite, Abdel-Khalek et al., 2019a, Jonauskaite, Abu-Akel et al., 2020a, Jonauskaite et al., 2024).

#### Experiment 4: Colour patches online

Similar to Experiment 3, we had pre-selected participants (i) who completed the survey in Arabic; (ii) whose native language was Arabic; (iii) who originally came from Saudi Arabia; (iv) who were not colour-blind by self-report, and (v) who took between 3 and 90 min to complete the survey. Furthermore, we selected participants based on their self-selected focal colours to ensure that they saw fitting colours for each colour category. To do that, we first converted participant colour choices from RGB to CIE *LCh* in two ways. For those who had performed the screen calibration procedure (Part 1), we could estimate the gamma curves of their screens from their responses and perform colour conversions. For those who had skipped the screen calibration procedure (Part 1), we assumed the standard 2.2 gamma (see also Epicoco et al., 2024) and used the ‘farver’ R package to make the conversions (Pedersen et al., 2024). Next, we excluded participants whose colour choices looked obviously wrong (e.g., chose a yellow hue for the category GREY), based on colour categorisation, derived from previous studies (Jonauskaite et al., 2016; Parraga & Akbarinia, 2016; see Table A2, OSM). Twenty-two participants (14 men, 0 other) were excluded in Experiment 4, leaving us with the total sample of 217 (92 men, one other) participants.

### Design and analysis

We employed a mixed design on our dataset of 220 data points per participant (11 colours x 20 emotions concepts). Participant were in one of the four between-subject conditions divided by two levels of set-up condition (laboratory vs. online) and two levels of colour presentation mode (terms vs. patches). Each participant associated 20 emotion concepts with 11 colours, making emotion and colour within-subject variables. We derived several dependent variables from the emotion concept selections, similar to previous studies (Jonauskaite, Abdel-Khalek et al., 2019a, Jonauskaite, Dael et al., 2019b, Jonauskaite, Abu-Akel et al., 2020a, Jonauskaite, Parraga et al., 2020b, Jonauskaite et al., 2021, 2024; Uusküla et al., 2023). When necessary, we controlled for family-wise error, arising from multiple comparisons, with the False Discovery Rate (FDR) correction (Benjamini & Hochberg, 1995). We analysed data using the R version 4.5.2 (R Core Team, 2025) using RStudio version 2026.1.0.392 (Posit team, 2026).

#### Patterns of colour-emotion associations

We created the patterns of colour-emotion associations by coding for the presence of each colour-emotion association. More precisely, we gave a value of 1 (association present) to all emotion intensity ratings between 1 and 5 and a value of 0 (association absent) when no emotion intensity rating was present (i.e., emotion concept had not been chosen). Subsequently, we calculated the proportion of participants in each of the four conditions who associated each colour with each emotion concept. The proportions varied from 0 (none associated) to 1 (everyone associated). These proportions, calculated for 220 colour-emotion combinations, constituted the pattern of colour-emotion associations. We used Pearson correlations to correlate the four patterns of colour-emotion associations with each other. This approach gives an indication of how similar the patterns were, with correlations theoretically varying from −1 (completely opposite patterns) to 0 (no similarity) to 1 (identical patterns). In addition, we performed these correlations for each colour, separately, to identify any colour-specific effects.

#### Affective biases: valence, arousal, and power

To calculate the valence bias, we counted the number of emotion concepts (n_total_) that each participant associated with the given colour. Then, we calculated the number of positive (n_positive_) and negative (n_negative_) emotion concepts (see Table 2). Finally, we subtracted the number of negative emotion concepts from positive emotion concepts and divided the difference by the total number of associated emotion concepts (see Eq. 1). Formally, the calculation can be described as:

1 $$Valence\ bias=\frac{({n}_{positive}- {n}_{negative})}{({n}_{positive}+{n}_{negative})}$$

Here, the maximum number of *n*_*positive*_ = 10, *n*_*negative*_ = 10, and (*n*_*positive*_ + *n*_*negative*_) = 20 emotion concepts (see Fig. 1). Thus, the valence bias could vary between −1 and 1. The extreme negative bias (−1) indicates that a participant associated only negative emotion concepts while the extreme positive bias (1) indicates that a participant associated only positive emotion concepts with a given colour.

In analogy, we calculated arousal and power biases (see Eqs. 2 and 3), by exchanging positive and negative emotion concepts, with high and low arousal, and high and low power emotion concepts, respectively.

2 $$Arousal\ bias=\frac{({n}_{high\ arousal}- {n}_{low\ arousal})}{({n}_{high\ arousal}+{n}_{low\ arousal})}$$

3 $$Power\ bias=\frac{({n}_{high\ power}- {n}_{low\ power})}{({n}_{high\ power}+{n}_{low\ power})}$$

Arousal bias could vary from −1 and 1, respectively, indicating that a participant associated only low arousing or high arousing emotion concepts with a given colour. Power bias could vary from −1 and 1, respectively, indicating that a participant associated only low power or high power emotion concepts with a given colour.

We fitted three analogous linear mixed models for valence, arousal, and power biases, using the *afex* R package (Mixed Model Anova Table (Type 3 tests, S-method); Singmann et al., 2024). Our categorical predictor variables were set-up condition (laboratory or online) and colour presentation mode (terms or patches). Our outcome variables were valence bias, arousal bias, or power bias. Our random intercepts were colour and participant, included to account for the repeated-measures design.

Formally, the models can be described as:

$$\begin{array}{lc}Valence\ bias \sim\ Setup \times Colour\ presentation\ mode+(1|Colour)+(1|Participant)\\ Arousal\ bias\ \sim\ Setup \times Colour\ presentation\ mode+(1|Colour)+(1|Participant)\\ Power\ bias\ \sim\ Setup \times Colour\ presentation\ mode+(1|Colour)+(1|Participant)\end{array}$$

#### Broadness and emotion intensity

Broadness defines the number of emotion concepts each participant associated with a given colour. It was calculated by simply counting the associated emotion concepts (irrespective of their intensity). The possible values of broadness range from 0 (no emotion concept was chosen) to 20 (all emotion concepts were chosen). Emotion intensity refers to the intensity of emotion concepts that were associated with each colour. We took all the emotion concepts that each participant associated with each colour and averaged their intensities, arriving at possible emotion intensity values from 1 (lowest intensity) to 5 (highest intensity).

To test the dependency of broadness and emotion intensity on the set-up condition and colour presentation mode, we fitted two analogous linear mixed models, just like we did for the affective biases. Again, our predictor variables were set-up condition (laboratory or online) and colour presentation mode (terms or patches), while our random intercepts were colour and participant, included to account for the repeated-measures design. Our outcome variables were broadness or emotion intensity.

The models can be described as:

$$\begin{array}{lc}Broadness \sim Setup \times Colour\ presentation\ mode+(1|Colour)+(1|Participant)\\ Emotion\ intensity\ \sim Setup \times Colour\ presentation\ mode+\left(1|Colour\right)+\left(1|Participant\right)\end{array}$$

## Results

### Self-selected colours

In Table A1 (OSM), we display the descriptive statistics of participants’ self-selected colours in Experiment 4 in terms of hue, chroma, and lightness (visually displayed in Figs. A2–A4, OSM). Numerically, participant lightness choices were the narrowest for WHITE, spanning just 6% of available values, and the widest for BLUE, GREEN, and PURPLE, spanning about 80% of available values. Participant chroma choices were the narrowest for the achromatic colour categories (i.e., WHITE, GREY, and BLACK), spanning just under 10% of available values, and the widest for BLUE, spanning about 80% of available values, as well as PURPLE, GREEN, and PINK, all spanning around 60–65% of available values. Finally, participant hue choices were the narrowest for YELLOW and RED, spanning about 7–8% of available values, while participant hue choices were the widest for BLUE, spanning 29% of available values, as well as PINK and BROWN, both spanning about 23–24% of available values.

### Patterns of colour-emotion associations

Across all participants and conditions, the most frequent colour-emotion association was RED-love, chosen by 61.5% of participants. Other frequent associations were RED-anger (43%), YELLOW-pleasure (43%), YELLOW-joy (38%), YELLOW-amusement (37%), GREEN-relief (44%), BLUE-relief (37%), PINK-joy (44%), PINK-pleasure (39%), PINK-love (38%), PINK-admiration (37%), BROWN-disgust (38%), WHITE-relief (56%), BLACK-sadness (47%), and BLACK-pride (36%).

**Fig. 2:**
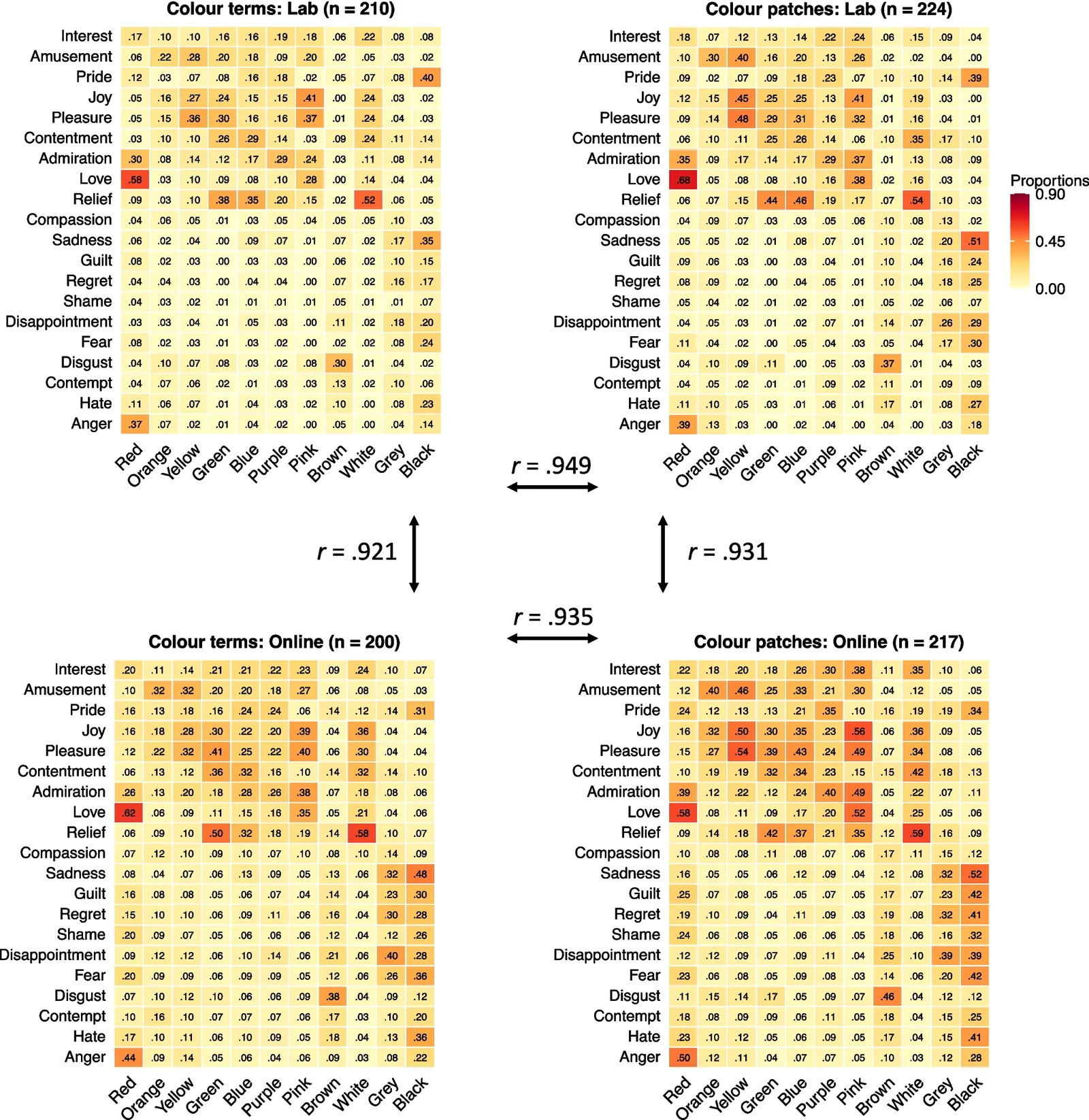
Colour-emotion association pattern correlations across the four experiments. Proportions indicate the number of participants choosing each colour-emotion association

The correlations between the patterns of colour-emotion associations were high and significant (see Fig. 2). They ranged between *r* =.92 and *r* =.95 (all *p*s ≤.001), indicating that participants chose nearly identical emotion associations, irrespective of whether the colours were presented in the laboratory or online, whether they appeared as colour terms or colour patches. For colour patches, they were either pre-selected (Experiment 2) or self-selected (Experiment 4). Likewise, similarly high correlations were obtained per colour, with correlations ranging between .815 (BLACK) to .991 (YELLOW; see Table 4 and Table A1, OSM).

**Table 4:** Colour-emotion association pattern correlations (Pearson *r* values) per colour across conditions

	Colour terms vs. Colour patches		Laboratory vs. Online	
Colour	Laboratory	Online	Colour terms	Colour patches
Red	.984	.965	.943	.920
Orange	.879	.872	.870	.853
Yellow	.982	.943	.915	.991
Green	.982	.971	.982	.968
Blue	.933	.897	.949	.937
Purple	.955	.952	.909	.978
Pink	.959	.976	.973	.981
Brown	.950	.952	.938	.936
White	.953	.979	.988	.943
Grey	.909	.962	.863	.901
Black	.947	.980	.815	.894

*Note*. In the first two columns, we used Pearson correlations to correlate patterns obtained when colours were presented as colour terms vs. as colour patches. We did so in two set-up conditions, once when presenting terms or patches in the laboratory (column one, Experiment 1 vs. Experiment 2) and once when presenting online (column two, Experiment 3 vs. Experiment 4). In the last two columns, we correlated patterns obtained in the laboratory vs. online. We did so for two colour presentation modes, once when presenting colour terms either in the laboratory or online (column three, Experiment 1 vs. Experiment 3) and once when presenting colour patches, either in the laboratory or online (column four, Experiment 2 vs. Experiment 4). All correlations were significant at *p* ≤.001, after correction for multiple comparisons

### Affective biases

#### Valence bias

**Fig. 3:**
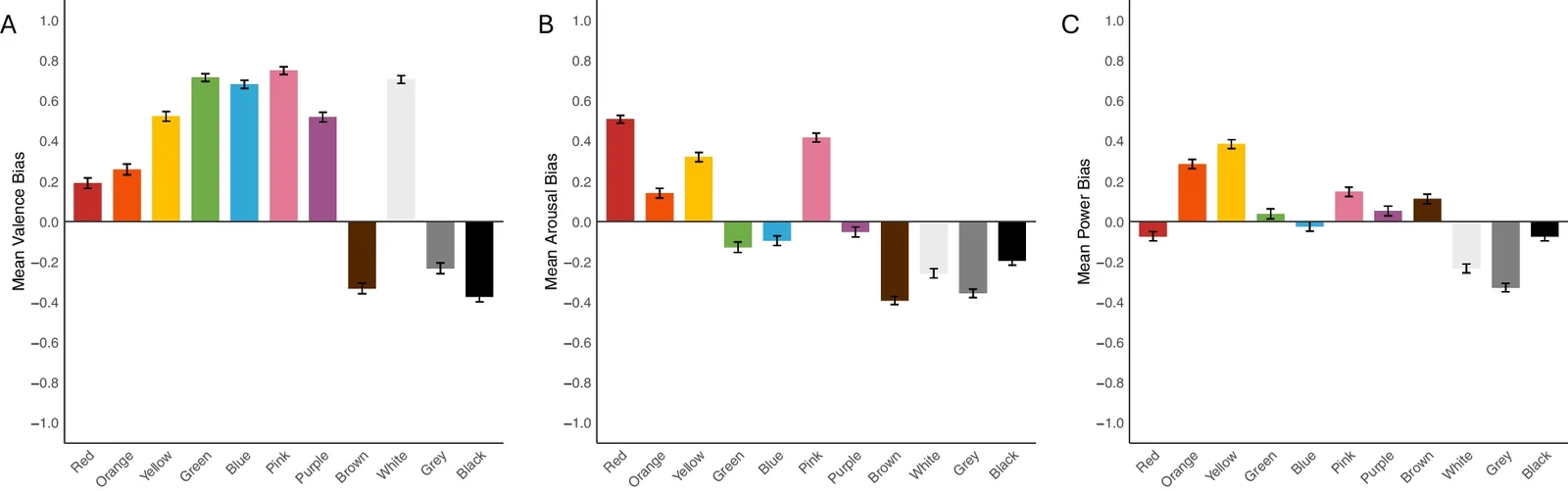
Bar charts of valence (**A**), arousal (**B**), and power (**C**) biases of all colour categories across the four experiments. Error bars indicate standard errors (SE) of the mean. Colours are for visualisation purposes only

On average and across all four experiments and colours, participants associated positively biased emotion concepts with colours, *t*(9360) = 37.29, *p* <.001, one-sample *t*-test. The valence bias varied by colour, *F*(10, 8500) = 356.0, *p* <.001, with RED, ORANGE, YELLOW, GREEN, BLUE, PURPLE, PINK, and WHITE biased towards positive emotion concepts and BLACK, GREY, and BROWN biased towards negative emotion concepts (see Fig. 3A).

A linear mixed-effects model was fitted to examine the effects of set-up, colour presentation mode, and their interaction on valence bias, with random intercepts for colour and participant. The full model explained significantly more variance than a null model containing only random effects, χ^2^(3) = 21.46, *p* <.001. The main effect of the set-up condition was significant, *F*(1, 847) = 21.46, *p* <.001, with participants associating more positive emotion concepts in the laboratory than online. In contrast, the main effect of colour presentation mode was not significant, *F*(1, 847) = 0.18, *p* =.674, demonstrating that participants associated emotion concepts of similar valence when colours were presented as terms and as patches. The interaction between the set-up condition and colour presentation mode was not significant, *F*(1, 847) = 0.02, *p* =.887.

#### Arousal bias

Emotion concepts that participants associated with colours were overall not biased in arousal, *t*(9360) = −0.96, *p* =.336. However, the arousal bias varied by colour, *F*(10, 8500) = 188.3, *p* <.001, with RED, ORANGE, YELLOW, and PINK biased towards high arousal emotion concepts and GREEN, BLUE, PURPLE, BROWN, WHITE, GREY, and BLACK biased towards low arousal emotion concepts (see Fig. 3B).

The linear mixed-effects model did not explain significantly more variance than the null model, χ^2^(3) = 1.49, *p* =.685. Analysis of fixed effects showed no significant main effects of the set-up condition, *F*(1, 847) = 0.02, *p* =.879, and the colour presentation mode, *F*(1, 847) = 1.44, *p* =.231, demonstrating that arousal bias of the associated emotion concepts did not differ between laboratory and online, nor between colour terms and colour patches. The interaction between the set-up condition and the colour presentation mode was not significant either, *F*(1, 847) = 0.03, *p* =.871.

#### Power bias

Emotion concepts that participants associated with colours were overall biased towards high power emotion concepts, *t*(9360) = 3.71, *p* <.001. The power bias also varied by colour, *F*(10, 8500) = 80.38, *p* <.001, with ORANGE, YELLOW, PINK, and BROWN biased towards high power emotion concepts and RED, WHITE, GREY, and BLACK biased towards low power emotion concepts (see Fig. 3C).

As for the arousal bias, the linear mixed-effects model did not explain significantly more variance than the null model containing only random effects, χ^2^(3) = 4.96, *p* =.175. Analysis of fixed effects showed no significant main effects of the set-up condition, *F*(1, 9347) = 1.54, *p* =.215, or the colour presentation mode, *F*(1, 9347) = 0.02, p =.902, nor a significant interaction between the set-up condition and the colour presentation mode, *F*(1, 9347) = 3.23, *p* =.072.

### Broadness and emotion intensity

On average, participants associated 2.68 emotion concepts (95% CI = [2.62, 2.74]) with each colour. The linear mixed-effects model explained significantly more variance than a null model containing only random effects, χ^2^(3) = 54.53, *p* <.001. There was a significant main effect of the set-up condition, *F*(1, 847) = 48.39, *p* <.001, with online participants choosing more emotion concepts, *M* = 3.27, 95% CI = [3.16, 3.38], compared to the participants in the laboratory, *M* = 2.12, 95% CI = [2.07, 2.17]. There was also a significant main effect of the colour presentation mode, *F*(1, 847) = 6.94, *p* =.009, with participants in the colour patches condition choosing a greater number of emotion concepts, *M* = 2.89, 95% CI = [2.81, 2.97], than those in the colour terms condition, *M* = 2.46, 95% CI = [2.37, 2.55]. However, the interaction between the set-up condition and the colour presentation mode was not significant, *F*(1, 847) = 0.32, *p* =.574.

Regarding emotion intensity, participants overall associated colours with emotion concepts of high intensity, *M* = 3.99, 95% CI = [3.97, 4.01]. The linear mixed-effects model was again significant, explaining more variance than the null model, χ^2^(3) = 32.45, *p* <.001. Analysis of fixed effects revealed a significant main effect of the colour presentation mode, *F*(1, 839.09) = 29.75, *p* <.001, with the colour terms condition producing higher intensity scores, *M* = 4.12, 95% CI = [4.09, 4.15], than the colour patches condition, *M* = 3.87, 95% CI = [3.84, 3.90]. The main effect of the set-up condition, *F*(1, 839.32) = 3.17, *p* =.076, and the interaction between the set-up condition and the colour presentation mode, *F*(1, 839.04) = 0.01, *p* =.929, were not significant.

## Discussion

We tested the consistency in colour-emotion associations as a function of colour presentation mode (colour terms vs. colour patches) and experimental setup (laboratory vs. online). Participants associated 11 colours (terms or patches) with 20 emotion concepts either in the laboratory or online. For terms, we used the basic colour terms in Arabic (Al-rasheed et al., 2011). For patches, we used pre-selected focal colours for the laboratory study (Al-rasheed, 2014) or participant self-selected representative colours online. Our results confirmed the basic predictions, namely, high degrees of similarity (*r* ≥.92) in the patterns of colour-emotion correspondences across the four study conditions.

For more concrete examples, participants associated diverse emotion concepts with colours (e.g., RED-love, WHITE-relief, BLACK-sadness), and those associations were many-to-many. In other words, one colour was associated with many emotion concepts, and one emotion concept was associated with many colours. For dimensional correspondences, participants associated (i) positive, high arousal emotion concepts with RED, ORANGE, YELLOW, and PINK; (ii) positive, low arousal emotion concepts with GREEN, BLUE, PURPLE, and WHITE; (iii) negative, low arousal emotion concepts with BROWN, GREY, and BLACK; (iv) high power emotion concepts with ORANGE, YELLOW, PINK, and BROWN; and (v) low power emotion concepts with RED, WHITE, GREY, and BLACK. Our results went in line with the general trends, reported in the recent systematic review (Jonauskaite & Mohr, 2025), showing that lighter and more chromatic colours are associated with more positive emotion concepts than darker and less chromatic colours, and warm colours (e.g., red, orange, yellow) are associated with more arousing emotion concepts than cool colours (e.g., blue, green).

For more detailed results, we replicated a high degree of similarity in the patterns of emotion associations with colour terms and colour patches in Arabic speakers in Saudi Arabia, previously reported from Swiss, Spanish, and Chinese participants (Jonauskaite, Parraga et al., 2020b, Jonauskaite et al., 2021, Wang et al., 2014; Xu et al., 2024). Previous studies also highlighted the universal aspect of colour-emotion correspondences, assessed across 30 nations, including Saudi Arabia (see Adams & Osgood, 1973; Jonauskaite, Abu-Akel, et al., 2020a). This high degree of similarity remained stable across testing modes, whether we tested participants online or in the laboratory. This observation implies that it did not matter whether individuals saw focal colours (laboratory) or had generated the most representative colour samples themselves (online). As a side note, the latter could vary tremendously, particularly in the cases of BLUE, GREEN, PINK, PURPLE, and BROWN. Moreover, it did not matter whether we looked at correspondences for discrete emotion concepts or affective dimensions. Thus, systematic colour-emotion correspondences are largely abstract and do not depend on having to see colours physically when taking such choices.

Despite these similarities, we also observed some peculiarities not reported before. For instance, 36% of our Saudi Arabian participants associated BLACK and PRIDE, which is not typically observed in Western samples (e.g., Fugate & Franco, 2019; Jonauskaite, Wicker, et al., 2019c; Zaikauskaite et al., 2023). Likewise, we found an association between RED and low power emotion concepts. RED has previously been linked to emotion concepts of high power and dominance such as *anger*, *hate*, and *passion* (Jonauskaite & Mohr, 2025; Mentzel et al., 2017). However, Saudia Arabian participants endorsed associations with *love* and *admiration* more strongly than with *anger*. Since both *love* and *admiration* are considered to have low power on the Geneva Emotion Wheel (Gillioz et al., 2016; Scherer et al., 2013), this likely explains the observed overall bias towards low power emotions (but see different types of love in Berscheid, 2010).

Furthermore, we did not replicate previous exceptions in term-patch correspondences for PURPLE and BLACK (Jonauskaite, Parraga et al., 2020b; Xu et al., 2024) in our Saudi Arabian participants, as emotion associations with colour terms and colour patches were similar for all 11 basic colour categories. That said, the current participants chose fewer but more intense emotion concepts for colour terms, suggesting that some behaviours differ when confronted with colour terms rather than colour patches (see similar results for intensity in Jonauskaite, Parraga et al., 2020b). Then, participants in the laboratory associated overall fewer but more positive emotion concepts with colours compared to the online participants. These differences might reflect the experimenter demand effects (e.g., Mummolo & Peterson, 2019; Rosenthal, 1976; Rosnow & Davis, 1977), which are more evident in laboratory experiments (White et al., 2018).

## Limitations and future directions

Although we tried to make the online and in-person conditions as comparable as possible, the two set-up conditions differed in terms of the testing location as well as inherent variabilities in screen calibrations, lighting conditions, and potentially other environmental factors. All of them are important for colour perception (Witzel, 2019). We tried to minimise this variability by instructing our online participants to adjust their screens in a standard way and self-calibrate the colour presentation (see Epicoco et al., 2024). Then, the laboratory experiments were conducted using paper and pencil and by showing physical colour patches. The online experiment was conducted on a computer, and participants self-selected colours, allowing us to get around some of the uncontrollable differences between screens.

Allowing participants to self-select the best examples for each colour category also meant that participants chose somewhat variable colours (see similar results in American English in Lindsey & Brown, 2014). On the one hand, RED, YELLOW, and achromatic colour categories were relatively uniform, as self-selected colours covered narrow hue ranges for RED and YELLOW, narrow lightness ranges for WHITE, and narrow chroma ranges for WHITE, GREY, and BLACK. These observations suggested that the mental representations of these categories are well defined and vary little between people. On the other hand, BLUE was the most varied colour category, with the widest ranges on all three colour dimensions. Other widely dispersed colour categories were GREEN and PURPLE on lightness and chroma, PINK on chroma and hue, and BROWN on hue. Knowing which colour categories vary the most, future studies could test for the importance of hue, lightness, and chroma variations on emotion associations within a single category.

Overall, our findings are encouraging for researchers interested in collecting data from diverse and geographically dispersed populations, as they suggest that online methods can yield results comparable to traditional highly controlled laboratory studies, and that they seem to hold in a different cultural context (namely, Saudi Arabia). Overall, this study opens new avenues for understanding the complex interplay between colour perception, conceptual knowledge, and emotional meaning. Future studies should continue to expand to other languages and cultures to further our understanding of colour-emotion correspondences.

## Supplementary Information

Below is the link to the electronic supplementary material.

Supplementary file1 (DOCX 422 KB)

Supplementary file2 (DOCX 1606 KB)

## Data Availability

The data are available at https://osf.io/5hc8j

## Footnotes

1 We also included *turquoise* in the survey, but it was not analysed here because it is not a basic colour term in Arabic (see Al-rasheed, 2014).

2 As in Experiment 3, we additionally presented the colour term *turquoise*, but did not analyse it here because it is not a basic colour term in Arabic and thus the data were not collected in the laboratory.

## Code availability

The analysis code has not been made available.
