# Supplementary material for: The stability of colour-emotion associations across colour presentation modes and experimental settings
Source: Psychon Bull Rev. 2026 Apr 20;33(5):146. doi: 10.3758/s13423-026-02881-z (PMC13661675; doi:10.3758/s13423-026-02881-z)
Supplement: Supplementary file 1 [file 13423_2026_2881_MOESM1_ESM.docx]

Table A 1. Descriptive statistics of the self-selected colours in Experiment 4 in the CIE *LCh* colour space.

| Colour category | Lightness | | | |  | Chroma | | | |  | Hue | | | |
| --- | --- | --- | --- | --- | --- | --- | --- | --- | --- | --- | --- | --- | --- | --- |
|  | Mean | 95% CI | Range width | Range limits |  | Mean | 95% CI | Range width | Range limits |  | Mean | 95% CI | Range width | Range limits |
| Red | 42.3 | [41.39-43.22] | 35.53 | 23.68- 59.21 |  | 81.36 | [79.89-82.84] | 49.84 | 54.71-104.55 |  | 37.44 | [36.89- 37.99] | 31.30 | 20.63- 51.93 |
| Orange | 64.83 | [64.14-65.53] | 33.66 | 49.56- 83.22 |  | 82.12 | [81.27-82.97] | 42.1 | 54.37- 96.47 |  | 56.48 | [55.52- 57.43] | 42.66 | 43.46- 86.12 |
| Yellow | 94.29 | [93.70-94.88] | 19.23 | 78.88- 98.11 |  | 89.73 | [88.84-90.62] | 47.63 | 49.28- 96.91 |  | 99.95 | [99.24-100.67] | 27.33 | 79.54-106.87 |
| Green | 58.96 | [56.80-61.11] | 78.79 | 15.24- 94.03 |  | 64.24 | [61.78-66.71] | 91.71 | 28.01-119.71 |  | 138.09 | [135.78-140.40] | 68.56 | 105.42-173.97 |
| Blue | 47.84 | [45.79-49.89] | 84.32 | 5.92- 90.25 |  | 48.72 | [45.51-51.94] | 112.41 | 21.40-133.81 |  | 262.94 | [259.80-266.08] | 105.06 | 202.10-307.16 |
| Purple | 35.61 | [34.07-37.15] | 74.60 | 5.85- 80.45 |  | 59.58 | [57.14-62.02] | 96.53 | 20.88-117.41 |  | 318.59 | [317.78-319.39] | 48.12 | 302.56-350.68 |
| Pink | 69.09 | [67.45-70.73] | 65.24 | 27.00- 92.25 |  | 47.65 | [45.23-50.07] | 83.53 | 10.97- 94.50 |  | 0.70 | [358.03- 3.36] | 87.15 | 317.51- 44.65 |
| Brown | 40.35 | [38.68-42.02] | 61.87 | 12.39- 74.26 |  | 47.82 | [46.03-49.61] | 67.01 | 13.32- 80.32 |  | 59.19 | [57.45- 60.93] | 83.57 | 23.82-107.39 |
| White | 99.82 | [99.72-99.92] | 6.25 | 93.75-100.00 |  | 0.22 | [0.06- 0.38] | 8.85 | 0.00-8.85 |  | - | - | - | - |
| Grey | 58.04 | [56.62-59.45] | 58.98 | 24.84- 83.81 |  | 0.65 | [0.49- 0.81] | 9.34 | 0.00- 9.34 |  | - | - | - | - |
| Black | 1.84 | [1.29- 2.39] | 24.84 | 0.00- 24.84 |  | 0.67 | [0.43- 0.91] | 9.81 | 0.00- 9.81 |  | - | - | - | - |

*Note*. We display means, 95% confidence intervals (CI) of the mean, range width (i.e., the difference between the highest and the lowest values) and range limits (i.e., the lowest and the highest values) for all colour categories on the three colorimetric dimensions. Possible values of lightness range from 0 to 100, for chroma – 0-140, for hue – 0°-360°. Since hue is a circular variable, measured in degrees, we present angular means, 95% angular CIs, linear range widths, and angular range limits. Hue is not meaningful for achromatic colours, this is why no values are presented for WHITE, GREY, and BLACK (see Figures A2-A4 for visual representations).

Table A 2. Inclusion criteria for self-selected colours, corresponding to each colour category.

| Colour category | *L* value (lightness) | *C* value (chroma) | *H* value (hue) |
| --- | --- | --- | --- |
| RED | 0-100 | ≥ 5 | 346°-72° |
| ORANGE | 0-100 | ≥ 5 | 20°-105° |
| YELLOW | 0-100 | ≥ 5 | 40°-130° |
| GREEN | 0-100 | ≥ 5 | 105°-220° |
| BLUE | 0-100 | ≥ 5 | 166°-346° |
| PURPLE | 0-100 | ≥ 5 | 220°-360° |
| PINK | 0-100 | ≥ 5 | 220°-72° |
| BROWN | 0-100 | ≥ 5 | 346°-130° |
| WHITE | 90-100 | ≤ 10 | 0°-360° |
| GREY | 10-95 | ≤ 10 | 0°-360° |
| BLACK | 0-30 | ≤ 10 | 0°-360° |

*Note*. Colour values are represented on the CIE *LCh* colour model. We inspired the colour categorisation from previous studies (Jonauskaite et al., 2016; Parraga & Akbarinia, 2016). Since participant colour choices varied substantially, we included also neighbouring colours as possible choices (e.g., for RED, we allowed choices falling into red and orange hues; for GREEN, we allowed choices falling to YELLOW-GREEN, GREEN, or GREEN-BLUE, etc. see (Jonauskaite et al., 2016). Therefore, the accepted hue, chroma, and lightness ranges were overlapping across the colour categories.
