# Supplementary material for: The stability of colour-emotion associations across colour presentation modes and experimental settings
Source: Psychon Bull Rev. 2026 Apr 20;33(5):146. doi: 10.3758/s13423-026-02881-z (PMC13661675; doi:10.3758/s13423-026-02881-z)
Supplement: Supplementary file 2 [file 13423_2026_2881_MOESM2_ESM.docx]

# Appendix

Figure A 1. Visual representation of the pre-selected focal colours, used in Experiment 2 (see values in Table 3).

*Note*. (A) CIE *Lab* model: a* x b* colorimetric coordinates. (B) CIE *LCh* model: lightness x chroma colorimetric coordinates. (C) CIE *LCh* model: hue x chroma. Hue is represented as a circular variable, measured in degrees, and chroma is displayed as a linear variable, extending from the centre (0) to the circumference of the circle (140).

Figure A 2. Distributions of self-selected colours along the CIE *Lab* a* x b* colorimetric coordinates. Stars represented pre-selected colours in Experiment 2.

Figure A 3. Distributions of self-selected colours along the CIE *LCh* lightness x chroma colorimetric coordinates. Stars represented pre-selected colours in Experiment 2.

**Figure A 4. Distributions of self-selected colours along the CIE *LCh* hue x chroma colorimetric coordinates (only chromatic colour categories are displayed).**

*Note*. Hue is represented as a circular variable, measured in degrees, and chroma is displayed as a linear variable, extending from the centre (0) to the circumference of the circle (140). The pre-selected colours used in Experiment 2 are marked with stars for comparison
